# Tofersen for SOD1 amyotrophic lateral sclerosis: a systematic review and meta-analysis

**DOI:** 10.1007/s10072-025-07994-2

**Published:** 2025-01-17

**Authors:** Abdullah Ashraf Hamad, Ibraheem M. Alkhawaldeh, Abdulqadir J. Nashwan, Mostafa Meshref, Yahia Imam

**Affiliations:** 1 Faculty of Medicine, Menoufia University, Menoufia, 32511 Egypt; 2 Faculty of Medicine, Mutah University, Al-Karak, Jordan; 3 Medical Research Group of Egypt, Negida Academy, Arlington, MA USA; 4 Nursing & Midwifery Research Department (NMRD), Hamad Medical Corporation, Doha, Qatar; 5 Department of Public Health, College of Health Sciences, QU Health, Qatar University, Doha, Qatar; 6 Department of Neurology, Faculty of Medicine, Al-Azhar University, Cairo, Egypt; 7 Neuroscience Institute, Hamad Medical Corporation, Doha, Qatar; 8 Weill Cornell Medicine, Doha, Qatar; 9 College of Medicine, Qatar University, Doha, Qatar

**Keywords:** Amyotrophic lateral sclerosis, ALS, Tofersen, *SOD1*, Antisense oligonucleotide

## Abstract

**Objective:**

Tofersen, an antisense oligonucleotide, has recently received FDA and EMA approval for treating amyotrophic lateral sclerosis (ALS) in adults with *SOD1* gene mutations. This systematic review and meta-analysis synthesized evidence on tofersen’s safety and efficacy in patients with *SOD1*-related ALS.

**Methods:**

A comprehensive search of three databases was conducted from inception through October 2024. Eligible studies included clinical trials, observational studies, and case studies. Meta-analyses were conducted using a random-effects model in RevMan.

**Results:**

Twelve studies involving 195 patients treated with tofersen met the inclusion criteria, comprising two randomized controlled trials (RCTs), five cohort studies, one case series, and four case reports. Tofersen demonstrated promising effects, notably reducing *SOD1* levels in cerebrospinal fluid and neurofilament light chain (NfL) in plasma, a biomarker strongly correlated with ALS progression and survival. Meta-analysis of RCTs showed a significantly lower rate of decline in ALS Functional Rating Scale-Revised (ALSFRS-R) scores from baseline in the tofersen group compared to placebo (SMD = 0.44, 95% CI [0.05 to 0.83], *P* = 0.03) and a significant reduction in the decline of predicted Slow Vital Capacity (*P* = 0.005). In a pre-post meta-analysis of five studies, a significant decrease in ALS progression rate (ALSFRS-R decline rate) was observed (MD = -0.28, 95% CI [-0.40 to -0.15], *P* < 0.0001). Reported adverse events were consistent with ALS progression or procedural effects.

**Conclusion:**

Current evidence suggests that tofersen effectively reduces SOD1 and NfL levels and slow disease progression in *SOD1* ALS, showing promise as a targeted therapeutic option.

## Introduction

Amyotrophic lateral sclerosis (ALS) is a debilitating neurodegenerative disease characterized by progressive muscle weakness and a drastically reduced life expectancy of about 2–5 years post-onset and 80% of patients being impacted within five years, primarily due to irreversible damage to upper and lower motor neurons. However, a small percentage of patients, approximately 10%, survive beyond 10 years after diagnosis [1, 2].

The identification of pathogenic variants in the Cu/Zn superoxide dismutase (*SOD1*) gene in 1993 marked a significant discovery in familial ALS, accounting for approximately 2% of sporadic cases and 11% of familial cases in Central Europe. This finding underscored the genetic underpinnings of ALS, further corroborated by subsequent genome sequencing studies. Notably, extensive genetic research has implicated several genes, including *SOD1*, *TARDBP*, *C9orf72*, and *FUS*, which collectively contribute to 60% of familial ALS cases and 11% of sporadic cases. *SOD1* mutations alone are responsible for 12% of familial ALS and 2% of sporadic cases, with over 230 different mutations identified, each presenting with diverse clinical manifestations [1, 3, 4].

Pathogenically, mutant *SOD1* proteins exhibit toxic gain-of-function properties implicated in neurotoxicity through mechanisms such as protein misfolding, proteasome dysfunction, oxidative stress, endoplasmic reticulum stress, disrupted axonal transport, inflammation, altered RNA processing, and mitochondrial dysfunction. Additionally, *SOD1* functions as a transcription factor for oxidative stress resistance genes, with RNA oxidation playing a significant role in neurodegeneration. Furthermore, astrocytes and oligodendrocytes derived from SOD1 mutation carriers can induce hyperexcitability and cell death in healthy motor neurons through both contact-mediated and soluble factors, highlighting glial involvement in ALS pathology [5].

Current therapeutic strategies for *SOD1*-related ALS aim to mitigate toxic protein aggregates and misfolded proteins through approaches such as lowering *SOD1* levels, RNA interference (RNAi), antibody therapies, and genome editing techniques. Pyrimethamine, for instance, has been shown to lower *SOD1* protein levels in ALS patients [6]. CRISPR and RNAi technologies, such as RfxCas13d and siRNAs packaged in AAV vectors, enhance blood-brain barrier permeability and effectively silence *SOD1* [7, 8]. Additionally, anti-*SOD1* nanobodies and the Derlin-1 epitope direct lysosomal degradation of misfolded *SOD1*. Gene editing techniques, including genome and base editing, have been successful in mouse models [1, 9, 10].

**Fig. 1:**
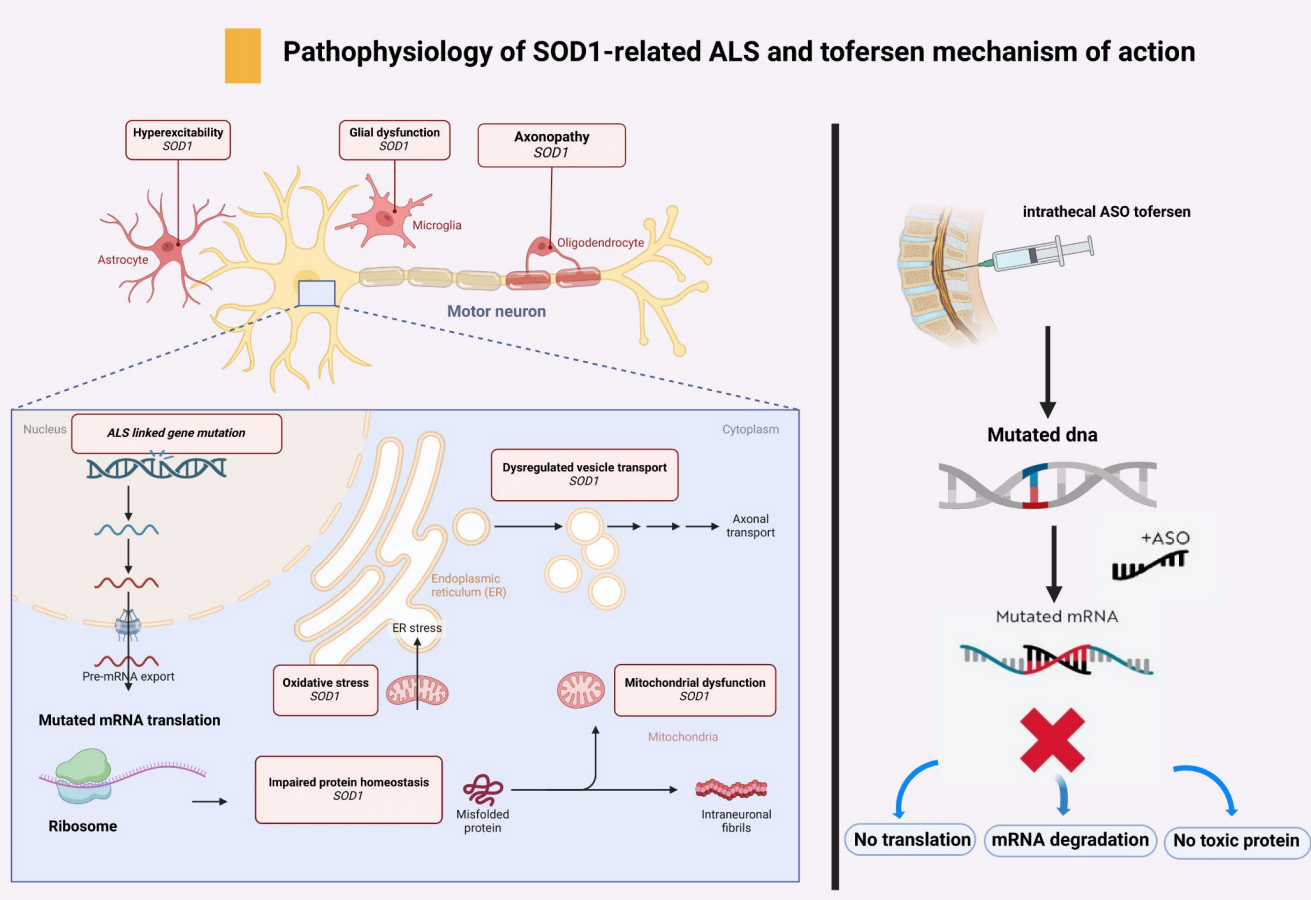
Pathophysiology of *SOD1*-related ALS and the mechanism of action of tofersen

Recent advancements include the Food and Drug Administration (FDA) and European Medicines Agency (EMA) approvals of antisense oligonucleotide (ASO) therapies such as tofersen, which reduces *SOD1* protein synthesis via RNase H-dependent degradation of *SOD1* mRNA when intrathecally administered (Fig. 1). Clinical trials have demonstrated tofersen’s ability to significantly reduce neurofilament light chain (NfL) levels in plasma, indicating potential disease-modifying effects, although improvements in ALS functional rating scale revised (ALSFRS-R) have varied [11].

The Open Label Extension (OLE) phase of the VALOR study revealed that early initiation of tofersen treatment correlated with a slower decline in ALSFRS-R scores over 52 weeks compared to delayed treatment initiation. However, approximately 7% of VALOR study participants experienced serious adverse events, including autoimmune reactions like myelitis, meningitis, and intracranial hypertension. These findings prompted the initiation of Early Access Programs (EAPs), providing further insights into tofersen’s safety and efficacy profile [4, 11].

Recent studies have expanded on these outcomes, detailing additional safety data and biomarker analyses, such as the detection of pleocytosis in the CSF of a significant proportion (60% and 73%) of patients, suggesting a potential extended immune system involvement [4, 12]. This systematic review and meta-analysis aimed to synthesize evidence on tofersen’s safety and efficacy in patients with *SOD1*-related ALS.

## Methods

### Data sources, searches, and study selection

This study adhered to the Preferred Reporting Items for Systematic Reviews and Meta-Analyses (PRISMA) guidelines [13]. The protocol for this systematic review and meta-analysis was registered in PROSPERO (CRD42024548491). A comprehensive search was conducted on PubMed, Web of Science, and Scopus databases from their inception until May 12, 2024, and subsequently updated on October 20, 2024. The search terms used were: (tofersen OR Qalsody) AND (“Motor Neuron Disease” OR “Motor System Disease” OR “Lou Gehrig’s Disease” OR “Amyotrophic Lateral Sclerosis” OR “Lateral Sclerosis” OR ALS). To ensure the completeness of the bibliography, the reference lists of relevant articles that met the inclusion criteria were manually searched.

Clinical trials, observational cohort studies, and case studies reporting on the assessment of tofersen for *SOD1* ALS were eligible for inclusion. Patients diagnosed with ALS and confirmed to have a *SOD1* mutation were considered eligible. Narrative and systematic reviews, commentaries, conference abstracts, and protocols were excluded. A two-step eligibility assessment was conducted for all retrieved studies. Initially, abstracts were screened against the eligibility criteria, followed by a full-text assessment of identified articles to make the final decision.

### Quality assessment and data extraction

The risk of bias for each included study was assessed using appropriate tools based on the study design. Randomized trials were evaluated using the Cochrane risk-of-bias tool for randomized trials (RoB 2) [14], while observational studies were assessed using the NIH tool for observational pre-post studies [15]. Case reports underwent evaluation using the JBI tool for case reports, and case series were evaluated using the NIH tool for case series [15, 16]. To extract the relevant data, an online data extraction form was utilized. The extracted data encompassed the following information:

- Study characteristics (study design, setting, sample size, and main findings).
- Baseline characteristics of the study participants.
- Domains related to the risk of bias.
- Study outcomes.

Two authors independently performed the data extraction using a standardized form. In cases where the mean and standard deviation were missing, they were estimated from the median and interquartile range using the Wan method [17]. Additionally, if the data were reported as a curve, WebPlotDigitizer was employed to extract the necessary information.

### Outcomes of interest

The primary outcomes of interest in this review focused on assessing the safety and efficacy of tofesen 100 mg in patients with *SOD1* ALS. Efficacy outcomes included the measurement of several parameters, such as the change from baseline in *SOD1* protein concentration in cerebrospinal fluid (CSF), the decline on ALSFRS-R, and the concentrations of Serum NfL. Additionally, safety findings encompassed the assessment of adverse events reported during the administration of tofersen. These primary outcomes were selected to evaluate the therapeutic impact of tofersen on disease progression and patient well-being in *SOD1* ALS.

### Data analysis

The meta-analysis included both RCTs and three cohort studies. Data analyses were conducted using RevMan version 5.4.1 for Windows, employing a random-effects model for all outcomes. Changes in ALSFRS-R and Forced Vital Capacity (FVC) were pooled as standardized mean differences (SMD) between tofersen and placebo, with standard errors (SE), as one RCT reported data using least-squares means. A pre-post meta-analysis of the five studies compared the ALS progression rate (ALS-PR) before and after treatment with tofersen, using mean difference (MD) and standard deviation (SD). Pre-therapy ALS-PR was calculated by dividing (48 - ALSFRS-R at baseline) by disease duration in months. Post-therapy ALS-PR was calculated by dividing the difference in ALSFRS-R scores between the initial and final examinations by the treatment duration in months. When possible, subgroup analyses were conducted based on disease progression. The I-squared (I²) statistic was used to assess heterogeneity across studies.

## Results

### Characteristics of included studies

**Fig. 2:**
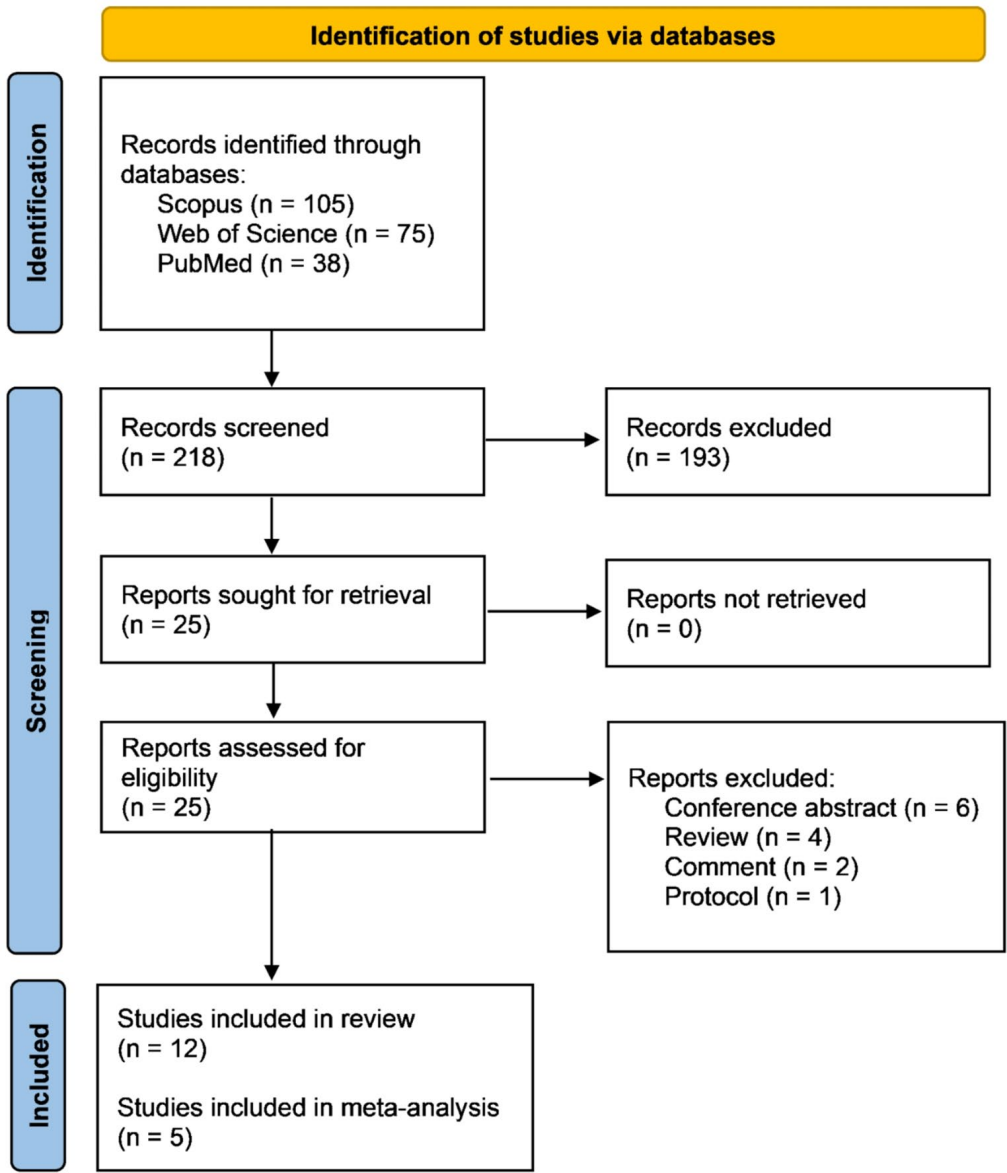
PRISMA flow diagram illustrating the study selection process

Our systematic database search identified a total of 218 articles. After title and abstract screening, 25 articles were selected for full-text screening. Ultimately, 12 articles met the inclusion criteria and were included in this review [4, 11, 12, 18–26] (Fig. 2). These articles comprised two randomized controlled trials (RCTs), five cohort studies, one case series, and four case reports. The majority of the studies were conducted in Germany (Table 1**)**. The overall sample size of ALS patients who received tofersen was 199; however, there was some overlap of patients across studies. Specifically, three patients from the Wiesenfarth study were reported in Meyer’s study and the patient reported by Forsberg was also enrolled in the VALOR study, resulting in a total of 195 unique patients receiving tofersen. Table 1 provides a summary of the characteristics of the included studies and their participants. The quality of the included studies was generally moderate (Supplementary file).

**Table 1:** Characteristics of the included studies and their participants

Study	Country	Study design	Sample size	males	Age, mean (SD)	Key findings
Miller 2022 [11]	10 different countries	Phase 3 RCT	Tofersen: 72 & placebo: 38	Tofersen: 43 & placebo: 19	Tofersen: 48.1 (12.6) & placebo: 51.2 (11.6)	Tofersen reduced concentrations of SOD1 in CSF and NfL in plasma, but did not significantly improve the disease progression and was associated with adverse events.
Miller 2020 [20]	6 different countries	Phase 2 ascending-dose RCT	Tofersen: 38 & placebo: 12	Tofersen: 21 & placebo: 7	Tofersen: 48.32 (10.82) & placebo: 49.2 (11.0)	CSF SOD1 concentrations decreased at the highest concentration of tofersen and adverse events were common.
Wiesenfarth 2024 [4]	Germany	Multicenter observational cohort	24	12	51.8 (15.4)	There was reduction of NfL serum levels, and pNfH CSF levels. adverse events were presents in some patients.
Sabatelli 2024 [12]	Italy	Multicenter observational cohort	17	12	58.6 (6.8)	During treatment, a significant statistical change was observed in the disease progression rate compared to the pre-treatment period.
Meyer 2024 [24]	Germany	Multicenter observational cohort	16	7	53 (9.3)	ALS progression rate was reduced by 25%. ALSFRS-R was increased in 7 patients. Most patients reported high treatment satisfaction.
Weishaupt 2024 [18]	Germany	Multicenter observational cohort	11	4	54.4	Tofersen decreased serum NfL in both homozygous and heterozygous patients.
Vinceti 2024 [19]	Italy	Multicenter observational cohort	10	5	Median = 58.6	Total selenium and key species increased significantly post-intervention.
Meyer 2023 [23]	Germany	Case series	6	2	53.3 (7.5)	There was reduction of NfL in both serum and CSF in all patients.
Gianferrari 2023 [22]	Italy	Case report	2	2	1st patient: 61 & 2nd patient: 53	There was a reduction in the ALSFRS-R decline in both patients after starting on tofersen administration
Reilich 2024 [25]	Germany	Case report	1	-	56	The study reports a case of myelitis as a side efect of tofersen therapy
Vidovic 2024 [26]	Germany	Case report	1	0	39	Tofersen treatment was associated with mild pleocytosis, as well as increased protein and albumin concentration in CSF, with no clinically significant adverse events.
Forsberg 2024 [21]	Sweden	Case report	1	1	In 30s	After four years on tofersen, the patient showed significant reduction in toxic SOD1 species and improved ALSFRS-R decline course with active social lifestyle.

*Abbreviations ALS*, Amyotrophic Lateral Sclerosis; *ALSFRS-R*, ALS Functional Rating Scale–Revised; *CSF*, Cerebrospinal Fluid; *NfL*, Neurofilament Light Chain; *pNfH*, Phosphorylated Neurofilament Heavy Chain; *SOD1*, Superoxide Dismutase 1

### Efficacy outcomes

Tofersen treatment demonstrated a reduction in concentrations of *SOD1* in CSF in both included RCTs compared to placebo [11, 20]. The geometric ratio to baseline of *SOD1* concentration in CSF was 0.65 and 0.70 in the tofersen groups, compared to 0.98 and 1.2 in the placebo group at the end of the RCTs [11, 20]. Additionally, there was a significant decrease in plasma NfL concentration across all the studies measuring it.

**Fig. 3:**
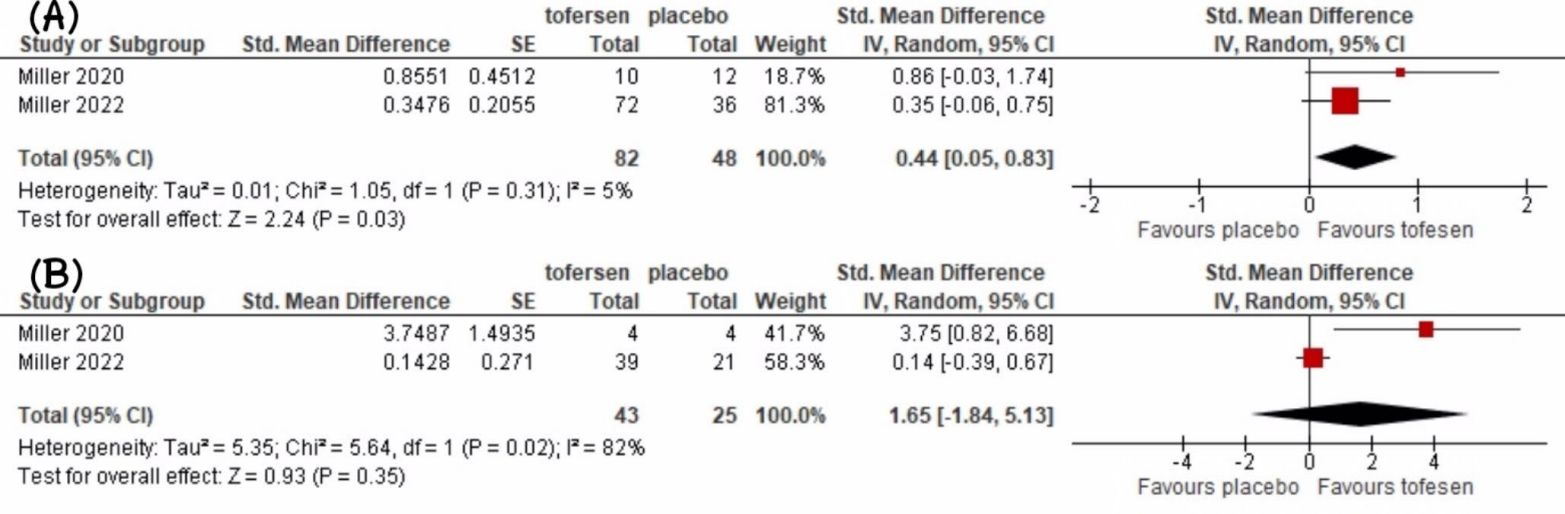
Meta-analysis forest plots; (**A**) Overall comparison of tofersen vs. placebo in terms of ALSFRS-R change, (**B**) Subgroup analysis for fast-progressing patients regarding ALSFRS-R change

**Fig. 4:**
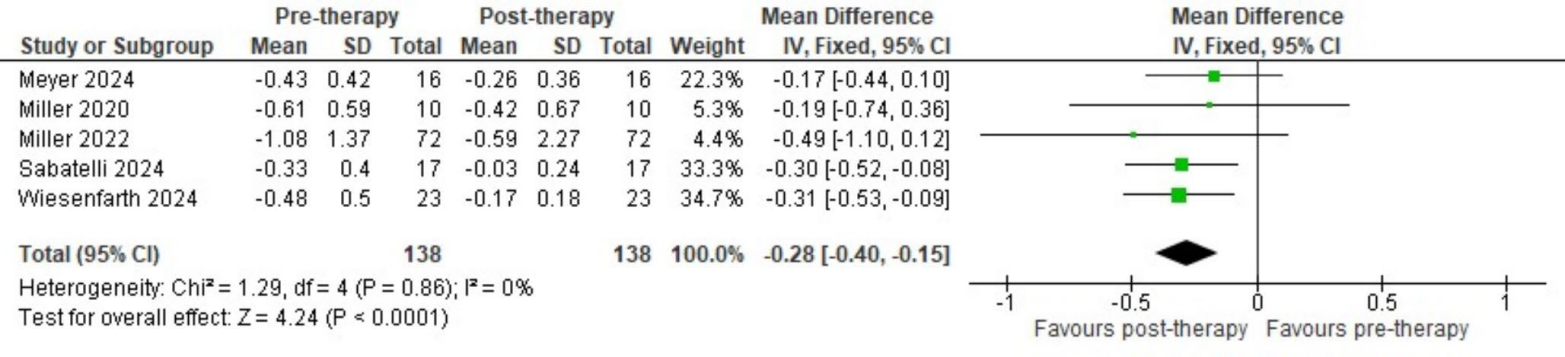
Pre-post meta-analysis comparing ALS-PR scores before and after tofersen treatment

**Fig. 5:**
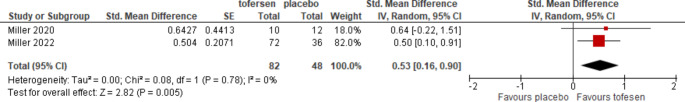
Meta-analysis forest plot comparing overall tofersen vs. placebo in terms of SVC

Regarding functional outcomes, a significant difference was observed between tofersen and placebo in terms of the change in ALSFRS-R from baseline (SMD = 0.44, 95% CI [0.05 to 0.83], *P* = 0.03) (Fig. 3**)**. However, subgroup analysis for fast-progression patients did not show a significant difference (*P* = 0.35) (Fig. 3**)**. Pre-post meta-analysis on patients receiving tofersen showed a significance reduction in ALS-PR favouring post therapy (MD = -0.28, 95% CI [-0.40 to -0.15], *P* < 0.0001) (Fig. 4**)**. Furthermore, there was a significant difference in the decline of the percentage of predicted SVC between tofersen and placebo (SMD = 0.53, 95% CI [0.16 to 0.90], *P* = 0.005), favouring tofersen **(**Fig. 5**)**.

These improvements in functional status were also observed in the cases reported by Gianferrari and Forsberg [21, 22]. Additionally, six patients in Wiesenfarth’s study and seven in Meyer’s study showed an increase in ALSFRS-R scores following tofersen therapy [4, 24]. Meyer et al. also evaluated patient-reported outcomes using the MYMOP (Measure Yourself Medical Outcome Profile), which indicated improved symptom severity in 10 patients and a perceived partial response in 6 patients [24].

Safety outcomes.

Overall, the observed adverse events were consistent with the natural progression of ALS or the treatment procedure. In the VALOR study, 7% of patients receiving tofersen experienced a total of eight neurologic serious adverse events, including myelitis, chemical or aseptic meningitis, lumbar radiculopathy, increased intracranial pressure, and papilledema [11]. In the other RCT, the incidence of adverse events was comparable between the tofersen and placebo groups [20]. Among the 38 patients in the tofersen group, five patients reported serious adverse events, while two patients in the placebo group (out of 12) experienced serious adverse events [20]. Similarly, two out of 23 patients in the Wiesenfarth study reported serious adverse events [4]. The most reported adverse events in the Meyer’s study were headache and back pain, with no severe adverse events [24].

## Discussion

This systematic review and meta-analysis aimed to summarize the current evidence on efficacy and safety of tofersen for *SOD1* ALS. In terms of efficacy outcomes, Tofersen treatment resulted in a reduction in SOD1 concentrations in CSF compared to placebo in the included RCTs. This decrease in CSF SOD1 levels can be attributed to the drug’s mechanism of action in suppressing the *SOD1* gene [27]. ALS patients typically show increased levels of NfL due to axonal damage. However, treatment with tofersen led to a significant decrease in plasma NfL concentrations across all the studies, indicating a potential slowing of disease progression.

Functional outcomes were also assessed, and significant improvements were observed in ALSFRS-R decline rate scores after tofersen therapy compared to the pre-therapy period. ALSFRS-R scores were even stopped or increased in some treated patients [4, 24]. This improvement can enhance the quality of life for ALS patients and reduce their need for assistance. Additionally, a significant difference was noted in the decline of the percentage of SVC between tofersen and placebo, which may delay respiratory failure, a common cause of death in ALS [28]. The VALOR and its Open Label Extension, as well as multiple case studies, have shown a significant slowdown in disease progression, as measured by ALSFRS-R scores, and a less rapid decline or stabilization of respiratory function.

The quality of the studies evaluating tofersen’s efficacy in ALS treatment were fair. While the studies provided valuable insights, it is important to note that the sample size in these trials was relatively small, which might limit the generalizability of the findings. However, despite these limitations, the observed effect sizes in the treated groups were notable, suggesting a potential therapeutic benefit of tofersen in ALS patients.

In recent research, the utilization of neurofilaments as biomarkers has gained attention in ALS studies. Neurofilaments are structural proteins found in neurons, and their levels in CSF or blood can reflect neuronal damage and disease progression [29]. Incorporating neurofilament measurements as biomarkers in ALS studies has provided valuable information on the disease’s pathophysiology and response to treatment [30]. By monitoring neurofilament levels, researchers can better understand disease progression and gauge the effectiveness of therapeutic interventions, as appeared in most of the included studies [23, 24].

Compared to prior ALS drugs such as riluzole and edaravone, tofersen has shown promising results in terms of disease progression and functional outcomes. While riluzole primarily targets glutamate release and Edaravone acts as a free radical scavenger [28], tofersen’s mechanism of action involves targeting the *SOD1* gene, which plays a role in protein production. This personalized approach based on genetic mutations offers a potentially more tailored treatment strategy for ALS. This personalized, mutation-based approach represents a significant step toward precision medicine in ALS therapies [31]. Furthermore, the use of biomarkers, including neurofilaments, and the consideration of disease progression in the study design have helped in assessing the efficacy of tofersen and monitoring its effects on patients with ALS.

Regarding safety outcomes, the most frequently reported side effects were related to disease progression or tofersen use. Common adverse effects included headache, injection site pain, fatigue, joint pain, pleocytosis (increased white blood cells in CSF), and muscle pain [11, 20]. In the VALOR study, a small percentage of patients receiving tofersen reported severe neurological adverse events, such as myelitis, meningitis, increased intracranial pressure, and papilledema. However, most of these events resolved without discontinuing the medication, although some required additional treatments such as glucocorticoids and plasma exchange. Other cases of myelitis and weakness were also reported, but the exact mechanism behind these neurological symptoms remains unclear [4, 25]. It is suggested to monitor CSF cell counts and total protein levels as a potential cause of transient weakness [25]. Another study reported two cases of autoimmune myeloradiculitis and lower limb weakness, both of which resolved clinically [4].

Our study represents the first systematic review and meta-analysis on tofersen in patients with ALS, providing a comprehensive summary of the available evidence on this topic. We strictly followed preferred guidelines for conducting systematic reviews and meta-analyses, ensuring the reliability and validity of our findings. However, a limitation of our study is that all the reported studies included in our analysis were conducted in Europe or the USA, which highlights the need for further research in different geographical regions to examine the generalizability of the findings to other racial and ethnic groups. Another limitation is the small sample sizes in most of the included studies, which may limit the statistical power and generalizability of the results. Also, pre post meta-analysis is not considered a highly reliable analysis, with a chance of the effect of confounding factors [32]. Further observational studies on patients receiving tofersen will strengthen the current literature and deepen our understanding of tofersen’s effects. Additionally, ongoing monitoring of post-marketing adverse events associated with tofersen remains crucial.

## Conclusion

In conclusion, the current evidence for tofersen in *SOD1* ALS demonstrates promising results, with significant reductions in CSF *SOD1* concentrations and plasma NfL levels, along with a slowing of disease progression as measured by ALSFRS-R. However, it is essential to acknowledge the limitations of existing studies, such as small sample sizes and limited RCT data. Given the challenges of conducting large RCTs in rare diseases like *SOD1* ALS, additional observational studies and registry-based data are crucial to further validate the safety and efficacy of tofersen. These alternative study approaches are particularly relevant for orphan and ultra-orphan diseases, where regulatory flexibility and innovative study designs can provide meaningful evidence outside traditional large RCT frameworks.
